# Radiomics‐Assisted Preoperative Subtyping of Adult‐Type Diffuse Gliomas: A Retrospective Study Aligned With the WHO 2021 Classification

**DOI:** 10.1002/cns.71225

**Published:** 2026-10-23

**Authors:** Yingzhun Liang, Jiawei Shi, Chongshun Zhao, Jiajia Liu, Ke Li, Jun Yang, Peiheng Ma, Hui Qiao, Wei Zhang, Xing Fan

**Affiliations:** ^1^ Beijing Tiantan Hospital Capital Medical University Beijing China; ^2^ Beijing Neurosurgical Institute Capital Medical University Beijing China

**Keywords:** adult‐type diffuse glioma, machine learning, preoperative subtyping, radiomics

## Abstract

**Background:**

Optimal surgical management of adult‐type diffuse gliomas (ADGs) varies substantially by subtype, making accurate preoperative subtyping crucial for individualized surgical planning. This study aimed to develop a radiomics‐based model for preoperative subtyping of ADG in accordance with the 2021 World Health Organization (WHO) Classification of Central Nervous System Tumors.

**Methods:**

A total of 382 patients with ADGs were enrolled and randomly assigned to a training set (*n* = 267) and an independent test set (*n* = 115). Tumor masks were automatically segmented and refined by experienced neurosurgeons. Radiomics features were extracted from MRI after standardized preprocessing and dimensionality reduction. Fourteen machine learning classifiers were evaluated, and the best‐performing model was optimized and validated on the independent test set. Model performance was assessed using AUC, accuracy, recall, precision, F1 score, Kappa coefficient, and Matthews correlation coefficient.

**Results:**

After LASSO (Least Absolute Shrinkage and Selection Operator) regression, 50 key features were retained. Random Forest showed the best preliminary performance and was selected for optimization. On the independent test set, the optimized model achieved a macro‐average AUC of 0.9084, with favorable results across other metrics. Texture features contributed most strongly to prediction.

**Conclusion:**

This radiomics model enables robust preoperative ADG subtyping under the 2021 WHO classification and may support individualized surgical planning using routine MRI features. Owing to its retrospective single‐center design, multicenter prospective external validation is required prior to widespread clinical application.

## Graphical abstract

An MRI‐based radiomics model accurately differentiated adult‐type diffuse glioma (ADG) subtypes under the 2021 WHO classification. Using routine preoperative imaging, the Random Forest classifier achieved strong performance and may support noninvasive, individualized surgical planning.

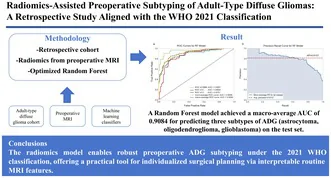

## 1 Introduction

Glioma is the most common primary malignant tumor of the central nervous system (CNS), with a global annual incidence of approximately 5–6 cases per 100,000 individuals [1]. It is characterized by marked heterogeneity, considerable prognostic variability, and complex management strategies, thereby imposing a substantial burden on public health [2, 3]. Among the diverse glioma spectrum, adult‐type diffuse gliomas (ADGs) constitute the majority of confirmed cases in the adult population, attracting widespread clinical and research interest from neurosurgeons and neuro‐oncologists [4]. The 2021 World Health Organization (WHO) classification of CNS tumors has revolutionized ADG diagnosis by establishing an integrated histomolecular diagnostic framework [5]. Specifically, ADGs are stratified into three subtypes according to isocitrate dehydrogenase (IDH) mutation and 1p/19q codeletion status: astrocytoma, IDH‐mutant; oligodendroglioma, IDH‐mutant and 1p/19q codeleted; and glioblastoma (GBM), IDH‐wildtype. These subtypes exhibit distinct biological behaviors, divergent therapeutic responses, and prognostic profiles, laying the foundation for personalized management [6, 7].

Neuropathological examination is the current gold standard for glioma classification and guides postoperative adjuvant therapy [8]. Regrettably, its unavailability before surgery restricts the incorporation of subtype information into initial surgical planning. Meanwhile, as the understanding of glioma surgical management continues to evolve, surgical strategies have become differentiated by tumor subtype. For instance, oligodendroglioma typically exhibits favorable chemosensitivity, and extensive resection may provide limited additional survival benefits [9, 10, 11, 12]. Accordingly, for lesions involving eloquent areas, neurological function preservation is generally prioritized over aggressive resection. In contrast, extensive resection confers a substantial survival benefit in patients with GBM [13]. For astrocytoma, grade‐adapted surgical planning may be required. In summary, a non‐invasive and accurate preoperative classification method for ADGs is of great significance for optimizing individualized surgical decision‐making. Radiomics represents a promising solution to addressing this challenge.

Magnetic Resonance Imaging (MRI) is the cornerstone of preoperative glioma assessment, delivering detailed morphological, microstructural, and textural information about tumors. As a high‐dimensional, quantitative radiological technique, radiomics enables high‐throughput extraction of subvisual features from MRI data and has been extensively integrated into glioma research [14, 15]. MRI‐based radiomics has been applied to the preoperative prediction of glioma grade, molecular subtypes, genetic alterations, treatment response, postoperative recurrence, survival outcomes, and seizure control, almost providing guidance throughout the entire course of glioma management [16, 17, 18, 19]. Nevertheless, the 2021 WHO classification has introduced substantial revisions to glioma categorization. For instance, some former adult gliomas are reclassified as pediatric‐type, and GBM is strictly restricted to IDH‐wildtype, with previously IDH‐mutant GBMs re‐designated as astrocytomas [5]. These changes render many research findings on ADGs outdated, highlighting the urgent need for updated investigations aligned with the current diagnostic framework [20]. To date, glioma‐related radiomics studies remain limited in both number and scope under the 2021 WHO classification [21]. In this study, we aimed to develop an MRI‐based radiomics model to differentiate the three newly defined ADG subtypes. The findings are expected to support preoperative individualized surgical planning and deepen our understanding of subtype‐specific imaging features.

## 2 Methods

### 2.1 Study Population

We retrospectively analyzed patients with glioma between October 2018 and June 2021. Inclusion criteria were: (1) newly diagnosed tumor; (2) diagnosis of ADG after re‐evaluation according to the 2021 WHO classification; (3) complete demographic and clinical data; and (4) available standardized preoperative brain MRI. Exclusion criteria were: (1) prior antitumor treatment; (2) history of another intracranial malignancy; and (3) poor imaging quality. A total of 382 patients were included and randomly assigned to a training set (*n* = 267) and an independent test set (*n* = 115) at a 7:3 ratio. Feature selection, model development, and optimization were performed using the training set only; the test set was reserved for final evaluation.

### 2.2 MRI Acquisition and Tumor Masking

All patients underwent preoperative MRI including T1‐weighted (T1W), T2‐weighted (T2W), contrast‐enhanced T1W (T1CE), and T2W FLAIR sequences on a 3.0‐T scanner (Siemens Healthineers, Erlangen, Germany) with a 32‐channel head coil. All MRI images were resampled to an isotropic voxel size of 1 × 1 × 1 mm^3^ using trilinear interpolation implemented in SimpleITK. Using DeepBraTumIA (Version 2.1.0, https://www.nitrc.org/projects/deepbratumia/) [22], T1CE images were registered to MNI152 space, and the other sequences were co‐registered to T1CE after skull stripping. Tumor masks included enhancing tumor and surrounding T2/FLAIR hyperintense regions. Automatically generated masks were independently reviewed and refined by two neurosurgeons (C. Zhao and P. Ma) with more than 5 years of experience; disagreements were resolved by a senior neurosurgeon with over 20 years of experience (W. Zhang).

### 2.3 Image Preprocessing and Feature Extraction

MRI data were preprocessed by intensity normalization, gray‐level discretization, and filter‐based image transformation. Voxel intensities were normalized to zero mean and unit variance, and outliers were removed. For each tumor region of interest (ROI), voxel intensities were normalized using *Z*‐score transformation based on the mean and standard deviation calculated within the tumor region. Tumor ROI intensities were discretized to 128 gray levels with a fixed bin width of 4. To capture multi‐scale texture features and expand the feature space, multiple combinations of low‐pass (L) and high‐pass (H) wavelet filters (LL, LH, HL, HH) were applied. Moreover, Laplacian of Gaussian (LoG) filters with sigma values of 1.0, 3.0, and 5.0 were employed to generate additional derived images.

Radiomics features were extracted from original and derived images according to Image Biomarker Standardization Initiative (IBSI) definitions. Features included 18 first‐order features, 14 shape features, and 68 texture features derived from Gray‐Level Co‐occurrence Matrix (GLCM), Gray‐Level Size Zone Matrix (GLSZM), Gray‐Level Run Length Matrix (GLRLM), and Gray‐Level Dependence Matrix (GLDM). Specifically, 18 first‐order and 68 texture features were extracted from eight images (one original, four wavelet sub‐bands, and three LoG‐filtered images) per sequence, yielding 2752 such features across the four MRI sequences. After combining with 14 shape features, a total of 2766 radiomics features were obtained per ROI.

### 2.4 Feature Selection

Feature selection was conducted via the Least Absolute Shrinkage and Selection Operator (LASSO)‐penalized multinomial logistic regression, which optimizes the multinomial negative log‐likelihood loss under an L1 regularization penalty. All radiomics features were first standardized using *Z*‐score normalization. The optimal regularization parameter (*λ*) was identified by 10‐fold cross‐validation in the training set. Cross‐validation and coefficient path plots were generated to visualize model deviance and coefficient shrinkage across different *λ* values. Features with non‐zero coefficients at the optimal *λ* were retained as the final radiomics signature for subsequent model construction.

### 2.5 Model Construction, Selection, and Optimization

Fourteen machine‐learning classifiers were compared for three‐class ADG classification: Random Forest (RF), Linear Discriminant Analysis (LDA), Naive Bayes (NB), Light Gradient Boosting Machine (LGBM), Extra Trees (ET), Gradient Boosting Classifier (GBC), CatBoost, Logistic Regression (LR), K Neighbors (KNN), SVM‐Linear Kernel (SVM), Quadratic Discriminant Analysis (QDA), Ada Boost, Decision Tree (DT), and Dummy Classifier. Initial model performance was assessed in the training set using 10‐fold cross‐validation, and training time was also recorded.

The optimal model was selected using a complementary assessment framework. First, classification accuracy was employed to reflect overall predictive correctness, providing an overview of the models' general effectiveness. Second, for evaluating the models' subtype‐specific performance. Recall and precision were used to quantify the models' sensitivity and positive predictive value for each class, and the F1‐score was calculated to provide a balanced summary of these two metrics. Third, to eliminate bias induced by fixed classification thresholds, receiver operating characteristic (ROC) curves were constructed using a one‐vs‐rest strategy. Additionally, macro‐averaged and micro‐averaged ROC curves were generated, with macro‐averaged ROCs averaging performance equally across subtypes and micro‐averaged ROCs weighted by subtype sample size to address class imbalance. Here, we used the area under the curve (AUC) of the macro‐averaged ROC curve as the primary evaluation metric. Finally, the Kappa coefficient and Matthews correlation coefficient (MCC) were used to assess agreement between predicted and actual labels beyond random chance and balanced performance under class imbalance.

Fourteen classifiers were preliminarily compared using their default PyCaret hyperparameter configurations without model‐specific tuning. Following the selection of the optimal classifier, dedicated tuning was conducted via Bayesian optimization implemented with the scikit‐optimize library. MCC was set as the optimization objective, with 50 search iterations paired with embedded 10‐fold cross‐validation. The tuned model was kept only when it surpassed the untuned counterpart. For each fold, accuracy, subtype‐specific AUC, recall, precision, F1‐score, Kappa coefficient, and MCC were calculated and reported as means ± standard deviations to assess model performance and stability.

### 2.6 Performance Evaluation of the Optimized Model

The test set, which was not used in any model training or optimization, was reserved for the final evaluation of the optimized model's generalization performance. The fully optimized model was subjected to a sequential multidimensional validation. First, to fully quantify the model's classification performance, subtype‐specific, macro‐averaged, and micro‐averaged ROC curves were generated. Subsequently, precision‐recall (PR) curves were generated to evaluate the trade‐off between precision and recall in the multi‐class setting, and average precision was computed to quantify positive predictive reliability. Furthermore, normalized confusion matrices were employed to visualize detailed classification outcomes and characterize inter‐subtype misclassification patterns, enabling the identification of common misclassification trends. Additionally, F1‐score, Kappa coefficient, and MCC were calculated to show the overall model performance. Finally, the permutation importance method was used to assess feature importance and identify the most influential radiomics features driving subtype prediction, thereby enhancing the model's interpretability.

### 2.7 Statistical Analysis

All statistical analyses were performed using R software (Version 4.4.2, https://www.r‐project.org/) and Python (Version 3.11.4, https://www.python.org). Data processing and visualization were primarily conducted using the “glmnet” package (Version 1.1‐37), “tidyverse” (Version 2.0.0), and “scikit‐learn” (Version 1.1.3) packages. Continuous variables were presented as mean ± standard deviation, while categorical variables were summarized as counts or percentages. The normality of continuous variables was assessed using the Shapiro–Wilk or Kolmogorov–Smirnov tests. Inter‐group comparisons were performed using the chi‐squared test, Fisher's exact test, Student's *t*‐test, and non‐parametric tests depending on variable type and distributions. The performance of machine learning models was evaluated using built‐in metrics from the PyCaret library (Version 3.0), including accuracy, AUC, recall, precision, F1‐score, Cohen's Kappa coefficient, and MCC. All *p*‐values were reported for two‐sided tests with results presented to four decimal places, and a *p*‐value < 0.05 was considered statistically significant.

## 3 Results

### 3.1 Patient Characteristics

Among the 382 patients enrolled in the final cohort (Table 1), 146 (38.22%) were diagnosed with astrocytomas, 126 (32.98%) with oligodendrogliomas, and 110 (28.80%) with GBMs. The overall cohort had a mean age of 45.58 ± 11.80 years, and comprised 239 males (62.57%) and 143 females (37.43%). The dataset was randomly divided into a training set (*n* = 267) and an independent test set (*n* = 115). No significant differences were observed between the training and test sets with respect to age, gender, tumor subtype, tumor grade, tumor volume, tumor diameter or tumor location (*p* > 0.05 for all), supporting the comparability and internal validity of both subsets for model development and independent performance evaluation.

**TABLE 1:** Clinical characteristics of the enrolled 382 patients.

Variables	Number	Overall *N* = 382^a^	Test *N* = 115^a^	Training *N* = 267^a^	*p*
Age	382	45.58 (11.80)	44.62 (11.33)	45.99 (11.99)	0.2982^c^
Gender	382				0.5723^b^
Male		239 (62.57%)	69 (60.00%)	170 (63.67%)	
Female		143 (37.43%)	46 (40.00%)	97 (36.33%)	
WHO 2021 Classification	382				0.9996^b^
A		146 (38.22%)	44 (38.26%)	102 (38.20%)	
GBM		110 (28.80%)	33 (28.70%)	77 (28.84%)	
O		126 (32.98%)	38 (33.04%)	88 (32.96%)	
Grade	382				0.3149^b^
2		149 (39.01%)	51 (44.35%)	98 (36.70%)	
3		102 (26.70%)	26 (22.61%)	76 (28.46%)	
4		131 (34.29%)	38 (33.04%)	93 (34.83%)	
Tumor volume	382	101.57 (75.93)	100.39 (69.86)	102.07 (78.53)	0.8084^c^
Maximum diameter	382	84.78 (23.92)	86.06 (23.15)	84.23 (24.27)	0.4767^c^
Lesion location	382				0.7494^b^
Frontal		130 (34.03%)	41 (35.65%)	89 (33.33%)	
Temporal		37 (9.67%)	11 (9.57%)	26 (9.74%)	
Insular		5 (1.31%)	3 (2.61%)	2 (0.75%)	
Parietal		20 (5.24%)	5 (4.35%)	15 (5.62%)	
Deep/Other		6 (1.57%)	2 (1.74%)	4 (1.50%)	
Multiregional		184 (48.17%)	53 (46.07%)	131 (49.06%)	

Abbreviations: A, astrocytoma, IDH‐mutant; GBM, glioblastoma, IDH‐wildtype; Maximum diameter, mm; O, oligodendroglioma, IDH‐mutant, and 1p/19q‐codeleted; Tumor volume, mL.

^a^ *n* (%); mean (SD).

^b^ Pearson's chi‐squared test.

^c^ Wilcoxon rank sum test.

### 3.2 Feature Selection

The extracted 2766 radiomics features were subjected to LASSO regression for dimensionality reduction. The LASSO coefficient profile curve is displayed in Figure 1a, and the cross‐validation curve of LASSO regression is presented in Figure 1b. As shown in the figure, when the penalty term *λ* takes the value between *λ*.min and *λ*.1se, ideal model fitting can be achieved. To preserve the maximal predictive radiomic information and attain the minimal cross‐validated classification error, we selected *λ*.min as the optimal regularization parameter. Ultimately, 50 radiomic features with non‐zero regression coefficients were retained to construct the radiomics signature for downstream modeling and analysis.

**FIGURE 1:**
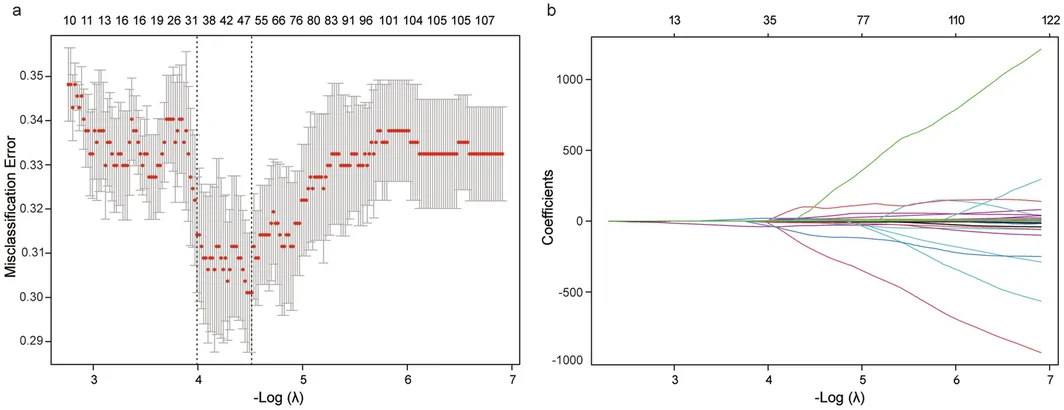
LASSO‐based feature selection for radiomics signature construction. (a) 10‐fold cross‐validation curve of the LASSO multinomial logistic regression model. The mean cross‐validated deviance is plotted against log(*λ*), with error bars indicating ±1 standard deviation. (b) LASSO coefficient profiles for all radiomic features. Each curve represents the trajectory of a single feature coefficient as a function of log(*λ*). LASSO, Least Absolute Shrinkage and Selection Operator.

### 3.3 Model Construction, Selection, and Optimization

Based on the 50 selected radiomics features, 14 mainstream machine learning classifiers were constructed to differentiate the three ADG subtypes. Their performance metrics are summarized in Table 2. Among all candidate models, the RF classifier exhibited the most robust overall performance, achieving the highest macro‐average AUC of 0.8549, along with well‐balanced results across other key evaluation metrics, including precision, recall, F1‐score, Kappa coefficient, and MCC. These findings collectively underscored the RF classifier's superior discriminative ability and stability in this three‐class classification task.

**TABLE 2:** Performance of machine learning classifiers in the classification.

Model	Accuracy	AUC	Recall	Precision	F1	Kappa	MCC	TT (Sec)
RF	0.6853	0.8549	0.6853	0.7092	0.6841	0.5263	0.5357	0.0690
LDA	0.6819	NA	0.6819	0.6944	0.6770	0.5199	0.5293	0.0110
NB	0.6779	0.8229	0.6779	0.7073	0.6710	0.5184	0.5346	0.0120
LGBM	0.6739	0.8364	0.6739	0.6853	0.6706	0.5102	0.5167	0.1210
ET	0.6708	0.8377	0.6708	0.6869	0.6689	0.5055	0.5122	0.0580
GBC	0.6707	NA	0.6707	0.6882	0.6652	0.5054	0.5158	0.2940
CatBoost	0.6707	0.8322	0.6707	0.6869	0.6670	0.5045	0.5125	11.3850
LR	0.6604	NA	0.6604	0.6760	0.6551	0.4875	0.4979	0.0300
KNN	0.6368	0.8036	0.6368	0.6547	0.6345	0.4530	0.4610	0.0310
SVM	0.6298	NA	0.6298	0.6409	0.6251	0.4379	0.4460	0.0180
QDA	0.5540	NA	0.5540	0.6172	0.5481	0.3094	0.3292	0.0120
Ada	0.5477	NA	0.5477	0.5778	0.5462	0.3116	0.3192	0.0460
DT	0.5241	0.6366	0.5241	0.5385	0.5220	0.2809	0.2855	0.0250
Dummy	0.3823	0.5000	0.3823	0.1466	0.2118	0.0000	0.0000	0.0170

*Note:* In binary classification settings, the AUC can be calculated using decision function scores for classifiers that do not natively output class probabilities (e.g., Ridge Classifier and linear SVM), as supported by the roc_auc_score implementation in scikit‐learn. However, in multi‐class classification tasks, AUC computation requires calibrated class probability estimates (predict_proba). As these linear classifiers do not provide native probability outputs in the multi‐class setting, AUC values are not applicable (NA) and are therefore not reported by PyCaret. For these models, classification performance should be primarily interpreted using accuracy, F1 score, Cohen's Kappa coefficient, and MCC.

Abbreviations: Ada, Ada Boost Classifier; AUC, area under the curve (all reported AUC values represent macro‐averaged AUC); DT, decision tree classifier; ET, extra trees; GBC, gradient boosting classifier; KNN, K neighbors classifier; LDA, linear discriminant analysis; LGBM, light gradient boosting machine; LR, logistic regression; MCC, Matthews correlation coefficient; NB, Naive Bayes; QDA, quadratic discriminant analysis; RF, random forest; SVM, SVM‐Linear Kernel.

To further enhance classification efficacy, the RF model was subjected to systematic hyperparameter tuning. Model generalizability was rigorously assessed via an independent outer loop of 10‐fold cross‐validation, with performance metrics for each fold detailed in Table 3. The model delivered outstanding discriminative power, with a mean macro‐average AUC of 0.8590, indicating a robust ability to distinguish among the three ADG subtypes. Meanwhile, it achieved stable, balanced mid‐range performance across core classification metrics, including accuracy, recall, precision, and F1‐score. Moreover, the moderate Kappa coefficient and MCC reflected good consistency and reliability beyond random chance. Furthermore, all key metrics exhibited relatively small standard deviations, which verified the model's stable performance across different data partitions and ruled out the risk of overfitting. Collectively, these findings support selecting the hyperparameter‐tuned, nested cross‐validated RF model as the final classifier, which was then rigorously evaluated on the independent test set to assess its real‐world generalization performance.

**TABLE 3:** Performance of the optimized RF classifier in 10‐fold cross‐validation.

Fold	Accuracy	AUC	Recall	Precision	F1	Kappa	MCC
0	0.7037	0.8626	0.7037	0.7212	0.7022	0.5574	0.5655
1	0.7778	0.8859	0.7778	0.7889	0.7814	0.6646	0.6660
2	0.6296	0.7839	0.6296	0.6429	0.6347	0.4410	0.4419
3	0.7037	0.8655	0.7037	0.7132	0.7068	0.5528	0.5539
4	0.7407	0.9385	0.7407	0.7407	0.7407	0.6095	0.6095
5	0.8148	0.8771	0.8148	0.8259	0.8185	0.7205	0.7220
6	0.5556	0.7950	0.5556	0.5247	0.5270	0.3306	0.3391
7	0.6538	0.8554	0.6538	0.6868	0.6504	0.4846	0.5023
8	0.6923	0.8623	0.6923	0.7295	0.6782	0.5478	0.5714
9	0.7308	0.8634	0.7308	0.7383	0.7327	0.5892	0.5905
Mean	0.7003	0.8590	0.7003	0.7112	0.6973	0.5498	0.5562
Std	0.0706	0.0415	0.0706	0.0783	0.0778	0.1057	0.1034

Abbreviations: AUC, area under the curve (all reported AUC values represent macro‐averaged AUC); MCC, Matthews correlation coefficient; RF, random forest; Std, standard deviation.

### 3.4 Model Classification Performance on the Independent Test Set

The optimized RF classifier was applied to the independent test set to validate its ability. The model achieved robust overall performance, with an accuracy of 0.7565, a macro‐average AUC of 0.9084, a precision of 0.7718, a recall of 0.7565, and an F1‐score of 0.7595. Notably, the Kappa coefficient (0.6305) and MCC (0.6337) indicated high agreement between predicted and actual subtypes, confirming the model's high predictive reliability beyond random chance in real‐world settings.

As presented in Figure 2a, the model's subtype‐specific discriminative performance showed marked heterogeneity on the independent test set. The one‐vs‐rest AUC values were 0.9557 for GBM, 0.8854 for astrocytoma, and 0.8701 for oligodendroglioma. The model yielded the highest AUC for glioblastoma, followed by astrocytoma and oligodendroglioma, indicating robust capacity to differentiate glioblastoma from the other two subtypes across classification thresholds. The PR curve (Figure 2b) achieved a micro‐averaged precision of 0.8157, substantially exceeding the no‐skill baseline, which reflects a favorable balance between precision and recall under multi‐class learning with moderately imbalanced data.

**FIGURE 2:**
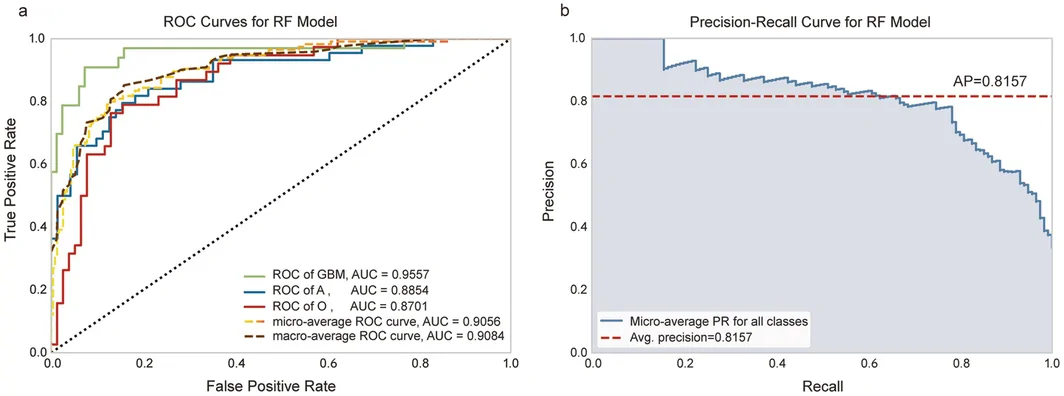
Discriminative performance of the optimized RF model. (a) ROC curves for the three subtypes classification. Together with the macro‐ and micro‐averaged ROC curves. The AUC is highest for GBM, followed by A and O. (b) Precision‐Recall (PR) curve of the RF model. The shaded area represents the micro‐averaged PR curve across all classes, and the AP (0.8157) summarizes the overall balance between precision and recall across different classification thresholds. A, astrocytoma; AP, average precision; AUC, area under the curve; GBM, glioblastoma; O, oligodendroglioma; PR, precision‐recall; RF, random forest; ROC, receiver operating characteristic.

The confusion matrix (Figure 3a) and classification report (Figure 3b) showed the model's performance under the single, fixed threshold selected for the multi‐class task. The model demonstrated high precision (0.9231) for identifying glioblastoma. Most misclassification events occurred between astrocytoma and oligodendroglioma. Specifically, 9 of 44 astrocytoma cases were misclassified as oligodendroglioma (20.45%); 8 of 38 oligodendroglioma cases were misclassified as astrocytoma (21.05%). This pattern aligns with their shared molecular background (IDH‐mutation) and overlapping imaging features, which are consistent with clinical observations. Feature importance analysis (Figure 4) revealed that top‐ranked predictive features were predominantly texture metrics derived from T1CE, T2W, and T2‐FLAIR sequences, including GLSZM and GLCM features. This result indicated that intratumoral heterogeneity contributes more to subtype differentiation than first‐order intensity or shape features, providing a biologically interpretable basis for the model's predictive capacity.

**FIGURE 3:**
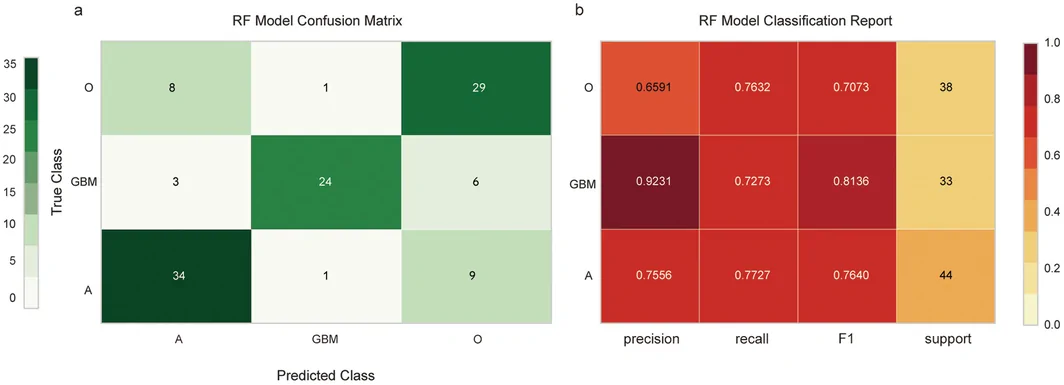
Classification performance of the optimized RF model. (a) Confusion matrix of predicted versus true adult‐type diffuse glioma subtypes. Normalized confusion matrix on the test set, showing the distribution of actual versus predicted labels for A, O, and GBM. Most GBM cases are correctly classified, whereas misclassifications mainly occur between the two IDH‐mutant subtypes (A and O). (b) Classification report of the RF classifier. It summarizes the precision, recall, and F1‐score for each subtype, demonstrating the best performance for GBM. A, astrocytoma; GBM, glioblastoma; O, oligodendroglioma; RF, random forest.

**FIGURE 4:**
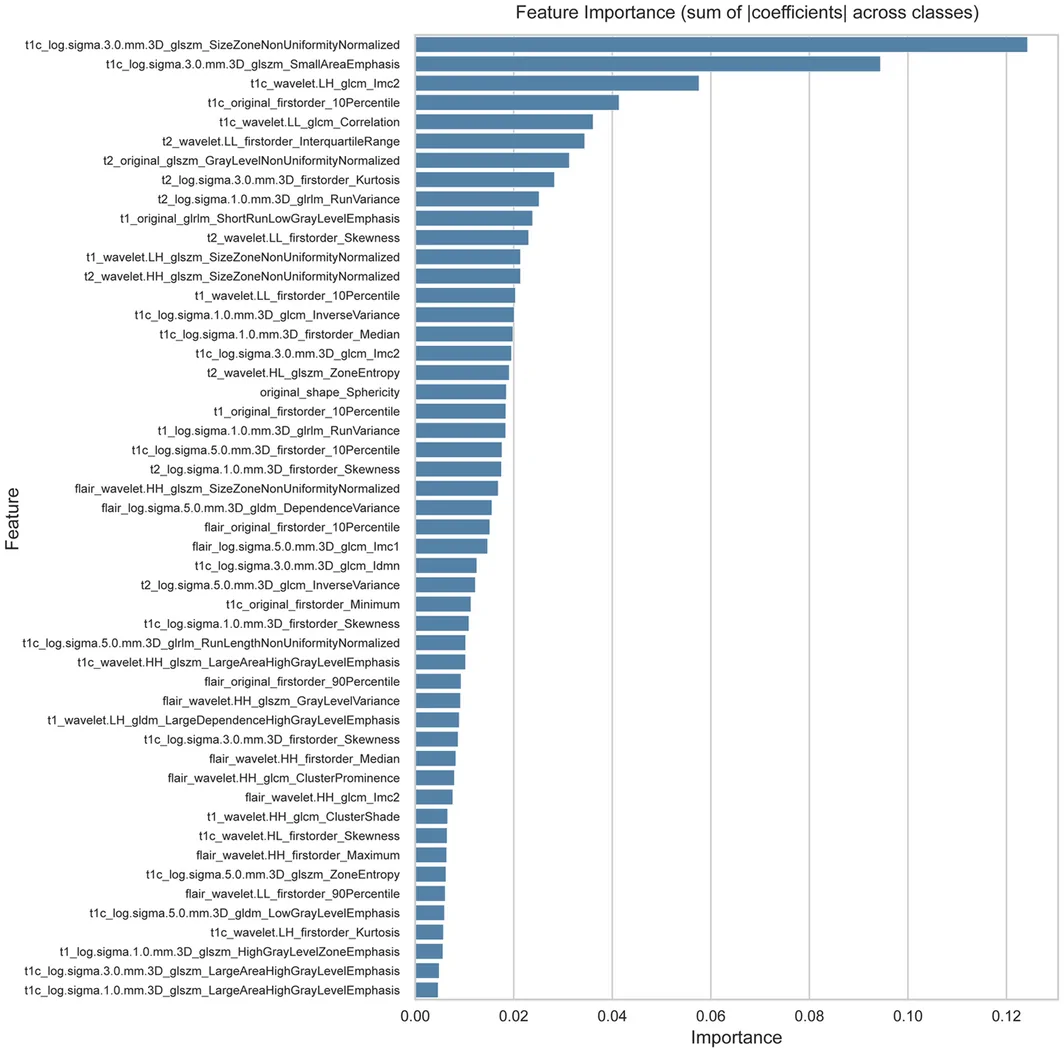
Feature importance of the RF radiomics model. Bar plot of the top‐ranked radiomic features according to their significance in the optimized RF model. The most influential predictors are predominantly texture features, especially GLSZM‐ and GLCM‐based descriptors from T1CE and T2W/FLAIR sequences. FLAIR, fluid‐attenuated inversion recovery; GLCM, gray‐level co‐occurrence matrix; GLSZM, gray‐level size zone matrix; RF, random forest; T1CE, T1‐weighted contrast enhanced; T2W, T2‐weighted.

## 4 Discussion

The current study developed and validated a radiomics‐based RF classifier for preoperative subtyping of ADGs according to the 2021 WHO classification. Our work can provide a robust, clinically translatable radiomics model for non‐invasive preoperative molecular subtyping of ADGs, thereby supporting the timely development of individualized surgical strategies for these patients.

From a clinical perspective, the application value of a predictive tool can be evaluated from three core dimensions. The first, and most fundamental prerequisite, is “significance”. The clinical significance of a predictive model is determined by whether its prediction target can directly guide clinical decision‐making and improve patient management. A model with excellent performance metrics but no practical clinical utility is of limited value for translational application. Take our previous explorations as an example; we once established a radiomics model to predict preoperative seizure occurrence in glioma patients [23]. However, because seizure is often the initial symptom prompting patients to seek medical attention, such a model offers negligible clinical guidance. In contrast, shifting the prediction target to postoperative seizure control would enable preoperative identification of high‐risk patients, facilitate tailored intraoperative and early postoperative antiepileptic interventions, and thus endow the model with substantial clinical relevance. Similarly, preoperative prediction of molecular alterations that primarily inform postoperative adjuvant therapy decisions may also show limited clinical significance [24, 25]. Notably, this does not diminish the value of these studies. Their strength lies not in clinical prediction but in uncovering intrinsic links between radiomic features and disease characteristics, and thus enriching the biological interpretation of imaging biomarkers.

In the current study, the preoperative ADG subtyping model demonstrates potential clinical value. It should be noted that radiomics‐based glioma subtyping had already achieved promising performance before the application of the 2021 WHO classification framework [26]. However, the 2021 WHO classification revolutionized the diagnostic paradigm of ADGs and rendered most pre‐existing radiomics models obsolete. In this context, our model fills the critical gap between advanced radiomics technology and the latest clinical classification framework, fully reflects practical needs, and enhances translational relevance. Its primary clinical significance is enabling preoperative, non‐invasive, and accurate differentiation of the three latest‐defined ADG subtypes, which directly translates into actionable, individualized surgical planning for clinicians. Concretely, for GBM, preoperative subtyping facilitates planning of maximal safe resection covering contrast‐enhancing lesions and accessible non‐enhancing tumor tissue, given the robust association between the extent of resection and survival outcomes [27]. For oligodendroglioma, function‐sparing resection protecting eloquent tissue is prioritized. For predicted astrocytoma, a grade‐adapted strategy balancing resection extent and neurological risk is recommended. Critically, the model uses only routine MRI sequences, enabling deployment in resource‐limited settings without advanced imaging and extending its clinical utility beyond tertiary centers. In addition, it should be emphasized that the model is not designed for standalone clinical use and must be interpreted together with multidisciplinary clinical information.

The second core dimension comes to “validity,” which reflects the model's ability to stably and accurately predict its target. In this study, we adopted a standardized, comprehensive radiomics workflow to ensure the validity of our model. First, for image processing, we adopted a hybrid strategy combining automated tumor segmentation with manual revision by experienced clinicians. This approach balanced the efficiency required for batch‐processing large cohorts with the accuracy of expert‐reviewed masks, laying a solid foundation for reliable feature extraction. Second, during the feature engineering phase, we strictly adhered to IBSI specifications for feature extraction and implemented Z‐score normalization followed by LASSO regression for dimensionality reduction. This step not only eliminated redundant and irrelevant features but also significantly reduced the risk of overfitting inherent to high‐dimensional data [28, 29]. Third, from a model‐construction perspective, we systematically evaluated the performance of 14 mainstream machine learning algorithms and selected the RF model to undergo further optimization via systematic hyperparameter tuning and cross‐validation. This could effectively balance bias and variance while reducing dependence on a single data split. The combined effect of the above strategies is fully reflected in the model's strong performance, especially given the complexity of three‐class classification compared to binary classification. During training, the model achieved a mean macro‐averaged AUC of 0.8590 via cross‐validation, with stable, balanced results across core classification metrics, indicating its reliable discriminative ability within the training cohort. More importantly, the model exhibited even better performance on the independent test set, with the macro‐averaged AUC increasing to 0.9084 and the Kappa coefficient and MCC rising to 0.6305 and 0.6337, respectively, suggesting substantial agreement. Additionally, the superior performance of the test set robustly confirms the absence of overfitting and demonstrates strong generalizability, which is a fundamental requirement for clinical translation.

To enable an objective assessment of the present model's performance and clinical translatability under the 2021 WHO criteria, Table 4 provides a cross‐study comparative summary against recent published studies [30, 31, 32, 33]. Against four existing three‐class prediction models developed under the 2021 WHO criteria, our study holds three distinct strengths. First, our prediction pipeline relies solely on routine structural MRI sequences without requiring advanced functional scans, histopathology whole‐slide images, or deep learning tools, which drastically reduce scanning expense, sampling risk, and computing load for routine clinical use. Second, our independent‐test macro‐AUC reaches 0.9084, outperforming the external validation metrics of all four prior models with far simpler imaging input. Third, we systematically screened 14 classifiers and conducted Bayesian hyperparameter optimization optimized for MCC to relieve multi‐class imbalance, while those previous studies only assessed a small batch of untuned algorithms. In summary, our model achieves better diagnostic accuracy alongside higher clinical accessibility and more robust modeling pipelines.

**TABLE 4:** Comparative overview of recent MRI radiomic studies for ADG subtyping under the 2021 WHO classification.

Study	Cohort size	Imaging input	Classifier selection	Model performance
Present study	382 (267 training/115 test)	Four routine sequences: T1, T1CE, T2, FLAIR	14 classifiers	Macro‐AUC: 0.9084; AP: 0.8157; per‐subtype AUC (A: 0.8854/O: 0.8701/GBM: 0.9557); ACC: 0.7565
Guo et al. [30]	894 (480 training/275 internal/139 external)	Five sequences: T1, T2, FLAIR, T1CE, DWI (ADC maps)	Single classifier only (RF)	Multiparametric model: internal AUC (A: 0.8181/O: 0.8502/GBM: 0.8524); external AUC (A: 0.7571/O: 0.7491/GBM: 0.7779); ACC internal 0.7382–0.8182, external 0.7266–0.7842
Pei et al. [31]	272 (166 training/106 validation)	Six sequences: T1, T2, FLAIR, T1CE, DSC‐PWI (rCBV, rCBF)	Single classifier only (RF)	Multimodal model: validation AUC (A: 0.778/O: 0.818/GBM: 0.861); ACC 0.726–0.755; comparable to combined model (all *p* > 0.05)
Liang et al. [32]	499 (306 training/132 test/61 external)	T1CE + T2‐FLAIR+whole‐slide pathology	6 classifiers compared (XGB, SVM, LR, KNN, RF, MLP)	Radiopathomics macro‐AUC: 0.847 (test)/0.858 (external), ACC: 0.742/0.705; radiomics‐only macro‐AUC: 0.786/0.788, ACC: 0.659/0.623; grading AUC: 0.849/0.855
Zhu et al. [33]	306 (180 training/78 internal/48 external)	Two sequences: 3D T1CE, T2‐FLAIR	6 classifiers compared (KNN, LightGBM, RF, SVM, SGD, XGB)	Combined model (XGB): internal macro‐AUC: 0.878, external macro‐AUC: 0.891; per‐subtype AUC internal (A: 0.866/O: 0.807/GBM: 0.934), external (A: 0.826/O: 0.864/GBM: 0.940)

Abbreviations: A, astrocytoma, IDH‐mutant; ACC, accuracy; ADC, apparent diffusion coefficient; ADG, adult‐type diffuse glioma; AP, average precision; AUC, area under the receiver operating characteristic curve; DSC‐PWI, dynamic susceptibility contrast perfusion‐weighted imaging; DWI, diffusion‐weighted imaging; FLAIR, fluid‐attenuated inversion recovery; GBM, glioblastoma, IDH‐wildtype; IDH, isocitrate dehydrogenase; KNN, k‐nearest neighbors; LightGBM, light gradient boosting machine; LR, logistic regression; MLP, multilayer perceptron; O, oligodendroglioma, IDH‐mutant and 1p/19q‐codeleted; rCBF, relative cerebral blood flow; rCBV, relative cerebral blood volume; RF, random forest; SGD, stochastic gradient descent; SVM, support vector machine; T1, T1‐weighted imaging; T1CE, contrast‐enhanced T1‐weighted imaging; T2, T2‐weighted imaging; WHO, World Health Organization; XGB, extreme gradient boosting.

The third core dimension is “interpretability.” Radiomics is well recognized for its strengths in high‐dimensional feature extraction and precise predictive performance, but its inherent “black‐box” nature has created a significant trust barrier in clinical application. A highly interpretable model can convert abstract prediction probabilities into understandable biological phenotypes for clinicians, thereby substantially enhancing their acceptance and willingness to adopt the model in routine practice. Moreover, exploring model interpretability can also deepen our understanding of disease biology.

In the present study, the model exhibited high precision for identifying GBM but showed partial misclassification between the two IDH‐mutant subtypes. This performance pattern is highly consistent with the well‐established clinical imaging features and biological behaviors of ADG subtypes. GBM typically presents with distinct morphological and structural hallmarks, like necrosis, ring enhancement, edema, and mass effect. These characteristics result in significant signal heterogeneity on MRI, making GBM easily distinguishable via texture and morphology‐based radiomics descriptors. In contrast, astrocytoma and oligodendroglioma share many conventional MRI characteristics, which limits the discriminative ability of radiomics models based solely on conventional MRI sequences [34].

Feature importance analysis further reinforced this interpretability, identifying GLSZM and GLCM descriptors from T1CE and T2W sequences as the top predictors. The GLSZM quantifies the size and gray‐level homogeneity of connected voxels with identical intensity [35]. Representative GLSZM metrics such as zone entropy can reflect heterogeneity in tumor cellular density and necrosis, directly mirroring GBM's disorganized growth with intermingled necrotic and hypercellular compartments, in contrast to the relatively uniform cellularity characteristic of IDH‐mutant subtypes. The GLCM encodes the spatial co‐occurrence of voxel intensity pairs to characterize local microstructural order [36]. GLCM‐derived correlation features on T1CE sequences correspond to microvascular proliferation and blood–brain barrier breakdown, which generate irregular contrast signals unique to GBM. In addition, our segmentation masks covered both enhancing lesions and T2/FLAIR peritumoral hyperintensity, enabling T2W‐derived GLSZM and GLCM features to further capture subtype‐specific edema profiles. The peritumoral zone of GBM consists of a mixture of vasogenic edema and infiltrating tumor cells, whereas IDH‐mutant astrocytomas are dominated by infiltrative tumor with mild vasogenic edema. Taken together, these high‐impact GLCM and GLSZM features reflect several critical subtype‐discriminating pathological characteristics of ADGs, including cellular density variation, necrosis, microvascular proliferation, and peritumoral edema. This means the model's predictions are not rooted in arbitrary mathematical parameters but in quantifiable radiological manifestations that correspond to biologically meaningful inter‐subtype differences. Such robust interpretability strengthens model reliability and provides new insights into imaging surrogates of ADG subtypes, supporting the clinical and translational application of radiomic tools.

Several limitations should be acknowledged. First, this is a single‐center study with MRI data acquired exclusively on Siemens 3.0T systems. Radiomics features, especially texture‐derived parameters, are highly sensitive to acquisition conditions including scanner vendor, field strength, reconstruction algorithms, and preprocessing pipelines. Internal cross‐validation cannot substitute multi‐vendor external testing, so the model's generalizability to external sites remains uncertain. Harmonization approaches such as ComBat correction combined with prospective multi‐vendor validation are required prior to clinical translation. Second, all performance assessments rely on retrospective data. Although the model achieves favorable subtyping accuracy, prospective confirmation remains absent regarding whether integration of this radiomic tool translates into measurable clinical benefits for patients with ADGs. Prospective clinical validation of its utility should be pursued in future investigations. Beyond subtyping, the model may be further extended to predict survival and seizure control, establishing a comprehensive, clinically relevant framework to optimize individualized glioma management.

## 5 Conclusion

The proposed radiomics‐based model exhibits robust performance for preoperative ADG subtyping aligned with the 2021 WHO classification, leveraging routine MRI‐derived interpretable features to offer a clinically practical tool for individualized surgical planning.

## Author Contributions

All authors contributed to the study conception and design. Material preparation, data collection, and analysis were performed by Yingzhun Liang, Jiawei Shi, and Chongshun Zhao. The first draft of the manuscript was written by Yingzhun Liang, and all authors commented on previous versions of the manuscript. All authors read and approved the final manuscript.

## Funding

This research was supported by the Beijing Research Ward Excellence Program (BRWEP2024W032040209, Xing Fan; BRWEP2024W032040201, Wei Zhang).

## Ethics Statement

This study was performed in line with the principles of the Declaration of Helsinki. Approval was granted by the Ethics Committee of Beijing Tiantan Hospital (Approval No.: KY2014‐002‐02), and written informed consent was obtained from all patients or their legal representatives.

## Conflicts of Interest

The authors declare no conflicts of interest.

## Data Availability

The data that support the findings of this study are available on request from the corresponding author.
